# Comprehensive Biostatistical Analysis of CpG Island Methylator Phenotype in Colorectal Cancer Using a Large Population-Based Sample

**DOI:** 10.1371/journal.pone.0003698

**Published:** 2008-11-12

**Authors:** Katsuhiko Nosho, Natsumi Irahara, Kaori Shima, Shoko Kure, Gregory J. Kirkner, Eva S. Schernhammer, Aditi Hazra, David J. Hunter, John Quackenbush, Donna Spiegelman, Edward L. Giovannucci, Charles S. Fuchs, Shuji Ogino

**Affiliations:** 1 Department of Medical Oncology, Dana-Farber Cancer Institute and Harvard Medical School, Boston, Massachusetts, United States of America; 2 Channing Laboratory, Department of Medicine, Brigham and Women's Hospital and Harvard Medical School, Boston, Massachusetts, United States of America; 3 Ludwig Boltzmann-Institute for Applied Cancer Research, and Applied Cancer Research – Institute for Translational Research, Vienna, Austria; 4 Department of Epidemiology, Harvard School of Public Health, Boston, Massachusetts, United States of America; 5 Department of Biostatistics, Dana-Farber Cancer Institute and Harvard Medical School, Boston, Massachusetts, United States of America; 6 Department of Biostatistics, Harvard School of Public Health, Boston, Massachusetts, United States of America; 7 Department of Pathology, Brigham and Women's Hospital and Harvard Medical School, Boston, Massachusetts, United States of America; Dresden University of Technology, Germany

## Abstract

**Background:**

The CpG island methylator phenotype (CIMP) is a distinct phenotype associated with microsatellite instability (MSI) and *BRAF* mutation in colon cancer. Recent investigations have selected 5 promoters (*CACNA1G*, *IGF2*, *NEUROG1*, *RUNX3* and *SOCS1*) as surrogate markers for CIMP-high. However, no study has comprehensively evaluated an expanded set of methylation markers (including these 5 markers) using a large number of tumors, or deciphered the complex clinical and molecular associations with CIMP-high determined by the validated marker panel.

**Metholodology/Principal Findings:**

DNA methylation at 16 CpG islands [the above 5 plus *CDKN2A* (p16), *CHFR*, *CRABP1*, *HIC1*, *IGFBP3*, *MGMT*, MINT1, MINT31, *MLH1*, p14 (*CDKN2A*/ARF) and *WRN*] was quantified in 904 colorectal cancers by real-time PCR (MethyLight). In unsupervised hierarchical clustering analysis, the 5 markers (*CACNA1G*, *IGF2*, *NEUROG1*, *RUNX3* and *SOCS1*), *CDKN2A*, *CRABP1*, MINT31, *MLH1*, p14 and *WRN* were generally clustered with each other and with MSI and *BRAF* mutation. *KRAS* mutation was not clustered with any methylation marker, suggesting its association with a random methylation pattern in CIMP-low tumors. Utilizing the validated CIMP marker panel (including the 5 markers), multivariate logistic regression demonstrated that CIMP-high was independently associated with older age, proximal location, poor differentiation, MSI-high, *BRAF* mutation, and inversely with LINE-1 hypomethylation and β-catenin (*CTNNB1*) activation. Mucinous feature, signet ring cells, and p53-negativity were associated with CIMP-high in only univariate analysis. In stratified analyses, the relations of CIMP-high with poor differentiation, *KRAS* mutation and LINE-1 hypomethylation significantly differed according to MSI status.

**Conclusions:**

Our study provides valuable data for standardization of the use of CIMP-high-specific methylation markers. CIMP-high is independently associated with clinical and key molecular features in colorectal cancer. Our data also suggest that *KRAS* mutation is related with a random CpG island methylation pattern which may lead to CIMP-low tumors.

## Introduction

Epigenetic aberrations are important mechanisms in human carcinogenesis [1], [2]. A number of tumor suppressor genes are silenced by promoter CpG island methylation [3], [4]. A subset of colorectal cancers exhibit widespread promoter methylation, which is referred to as the CpG island methylator phenotype (CIMP) [4]–[7]. CIMP-high colorectal tumors have been associated with older age, female sex, proximal location, mucinous and poor differentiation, microsatellite instability (MSI), *BRAF* mutation, high LINE-1 methylation level, wild-type *TP53*, stable chromosomes, and inactive WNT/β-catenin [8]–[23]. However, many of these features are interrelated, and thus, it is essential to analyze a large number of tumors by multivariate analysis to decipher the complex relations between CIMP-high and these clinical/tumoral variables.

There is considerable heterogeneity of tumors with regard to CpG island methylation, and not all CpG islands are methylated in a similar manner in colorectal cancer [15]. Thus, choice of CpG islands can substantially influence the features of CIMP. In fact, different CIMP panels used in various studies have caused considerable confusion [7]. Weisenberger et al. [15] have screened 195 CpG islands, and selected 5 loci (*CACNA1G*, *IGF2*, *NEUROG1*, *RUNX3* and *SOCS1*), which can serve as surrogate markers for CIMP-high. We have further validated the use of 8 markers (the above 5 plus *CDKN2A* (p16), *CRABP1* and *MLH1*) as a CIMP-high diagnostic panel [24]. However, no study has comprehensively compared these CIMP-high-specific CpG islands and other CpG islands using a large number of tumors.

In this study, we have assessed 16 CpG islands including the new 5 CIMP markers as well as MINT (methylated in tumor) markers and other CpG islands, utilizing hierarchical clustering analysis on a large number of colorectal cancers. We have also assessed the characteristics of CIMP-high tumors determined by a validated marker panel, and interactions of various clinical and tumoral factors by multivariate logistic regression analysis. This study provides the rationale for standardization of CIMP-high-specific methylation markers.

## Methods

### Study Group

We utilized the databases of two large prospective cohort studies; the Nurses' Health Study (NHS, N = 121,700 women followed since 1976) [25], [26], and the Health Professionals Follow-up Study (HPFS, N = 51,500 men followed since 1986) [26]. A subset of cohort participants developed colorectal cancer during prospective follow-up. Thus, these colorectal cancers represented a population-based, relatively unbiased sample (compared to a single or few-hospital-based sample). Previous studies on the cohorts have described baseline characteristics of cohort participants and incident colorectal cancer cases, and confirmed that our colorectal cancers were well representative as a population-based sample [25], [26]. Clinical information was obtained through chart review by physicians. We collected paraffin-embedded tissue blocks from hospitals where participants had undergone resections of primary colorectal cancers. Based on availability of adequate tissue specimens, a total of 904 colorectal cancer cases (406 from the men's cohort and 498 from the women's cohort) were included. Clinical characteristics of the cases are described in **Table 1** (on the left, under the column heading “All cases”). Among our cohort studies, there was no significant difference in demographic features between cases with tissue available and those without available tissue [26]. Most tumors have previously been characterized for statuses of MSI, CIMP, *KRAS*, *BRAF*, p53, β-catenin, LINE-1 methylation and 14 of the 16 methylation markers [19], [21], [24], [27]. However, none of our previous studies have comprehensively analyzed the 16 methylation markers in relation to each other, independent associations of CIMP with various clinical, pathological or tumoral molecular characteristics, or interactions of various factors on the associations with CIMP-high by comprehensive biostatistical methods. This study represents a unique novel study in term of 1) a large sample size; 2) the validated set of CIMP-specific methylation markers; 3) the number of other molecular events analyzed, including 8 CpG islands other than the CIMP-specific markers, MSI, *KRAS*, *BRAF*, p53, LINE-1 methylation and β-catenin; and 4) comprehensive statistical analyses including unsupervised hierarchical clustering, smoothing splines to assess nonlinearity, multivariate logistic regression, and stratified logistic regression. Thus, this study obtained novel data from the existing materials and database, analogous to novel studies using the well-described cell lines or mouse models. Informed consent was obtained from all study subjects. Tissue collection and analyses were approved by the Harvard School of Public Health and Brigham and Women's Hospital Institutional Review Boards.

**Table 1:** Clinical, pathologic and molecular characteristics of colon cancer cases according to CIMP status.

Clinical, pathological or molecular feature	All cases	CIMP-low/0	CIMP-high	P value
Total N	904	771	133	
Sex				0.003
Male	406 (45%)	362 (47%)	44 (33%)	
Female	498 (55%)	409 (53%)	89 (67%)	
Mean age±SD	66.3±8.4	65.7±8.5	69.4±6.9	<0.0001#
Tumor location∧				<0.0001
Proximal	386 (45%)	270 (37%)	116 (89%)	
Distal (excluding rectum)	282 (33%)	270 (37%)	12 (9.2%)	
Rectum	199 (23%)	196 (27%)	3 (2.3%)	
Tumor stage				<0.0001
I	203 (25%)	188 (28%)	15 (12%)	
II	261 (33%)	193 (29%)	68 (54%)	
III	229 (29%)	203 (30%)	26 (21%)	
IV	106 (13%)	90 (13%)	16 (13%)	
Tumor differentiation				<0.0001
Well/moderate	803 (91%)	710 (94%)	93 (70%)	
Poor	83 (9.4%)	43 (5.7%)	40 (30%)	
Mucinous component				<0.0001
0%	601 (66%)	545 (71%)	56 (42%)	
1–49%	188 (21%)	152 (20%)	36 (27%)	
≥50%	115 (13%)	74 (9.6%)	41 (31%)	
Signet ring cells				<0.0001
0%	844 (93%)	733 (95%)	111 (83%)	
≥1%	60 (6.6%)	38 (4.9%)	22 (17%)	
MSI				<0.0001
Non-MSI-high	748 (85%)	707 (95%)	41 (31%)	
MSI-high	127 (15%)	37 (5.0%)	90 (69%)	
Mean LINE-1 methylation (%)±SD	61.4±9.6	60.7±9.6	65.1±8.3	<0.0001#
*BRAF*				<0.0001
Wild-type	748 (87%)	697 (95%)	51 (40%)	
Mutated	113 (13%)	36 (4.9%)	77 (60%)	
*KRAS*				<0.0001
Wild-type	557 (63%)	445 (59%)	112 (85.5%)	
Mutated	326 (37%)	307 (41%)	19 (14.5%)	
p53*				<0.0001
Negative	506 (57%)	403 (53%)	103 (77%)	
Positive	386 (43%)	356 (47%)	30 (23%)	
β-catenin*				<0.0001
Inactive (score 0–2)	499 (64%)	403 (60%)	96 (91%)	
Active (score 3–5)	278 (36%)	269 (40%)	9 (8.6%)	

(%) indicates the proportion of cases with a specific clinical feature within each MSI/CIMP subtype.

∧ Proximal colon includes cecum to transverse colon, and distal colon includes splenic flexure to sigmoid colon.

* p53 and β-catenin status was determined by immunohistochemistry. Active β-catenin was defined as β-catenin score ≥3, where the β-catenin score was the sum of nuclear (0, 1+ or 2+), cytoplasmic (0, 1+ or 2+) and membrane (0 or 1+ if expression is lost) scores as originally described by Jass et al.[35].

# t-test assuming unequal variances.

CIMP, CpG island methylator phenotype; LINE-1, long interspersed nucleotide element-1; MSI, microsatellite instability; SD, standard deviation.

### Pathologic Examination, DNA Extraction and Sequencing of *KRAS* and *BRAF*

For all cases, pathologic features including tumor differentiation, mucinous features and signet ring cells were examined by a pathologist (S.O.). Poor differentiation was defined as the presence of <50% glandular area. Genomic DNA was extracted from paraffin tissue, and PCR and Pyrosequencing targeted for *KRAS* codons 12 and 13, and *BRAF* codon 600 were performed as previously described [28], [29].

### Microsatellite Instability (MSI) Analysis

MSI status was determined by the MSI panel including D2S123, D5S346, D17S250, BAT25, BAT26, BAT40, D18S55, D18S56, D18S67 and D18S487 (i.e., 10-marker panel) as previously described [24]. A “high degree of MSI” (MSI-high) was defined as the presence of instability in ≥30% of the markers.

### Real-time PCR (MethyLight) for Quantitative DNA Methylation Analysis

Sodium bisulfite treatment on DNA and subsequent real-time PCR (MethyLight [30]) was validated and performed as previously described [31]. We quantified DNA methylation in 5 CIMP-specific promoters (*CACNA1G*, *IGF2*, *NEUROG1*, *RUNX3* and *SOCS1*) and 11 other CpG islands [*CDKN2A* (p16), *CHFR*, *CRABP1*, *HIC1*, *IGFBP3*, *MGMT*, MINT1, MINT31, *MLH1*, p14 (*CDKN2A*/ARF), and *WRN*]. *COL2A1* (the collagen 2A1 gene) was used to normalize for the amount of template bisulfite-converted DNA [31]. Primers and probes were previously described [15], [27], except for *IGFBP3*, p14 and *WRN*: IGFBP3-F, 5′-G***T***T ***T***CG GGC GTG AG***T*** ACG A-3′ (Genbank No. M35878, nucleotide Nos. 1692-1710); IGFBP3-R, G***AA*** TCG ***A***CG CA***A*** ** ***A***CA CG***A*** CT***A*** C(nucleotide Nos. 1789-1810) and IGFBP3-probe, 6FAM-***T***CG G***T***T G***T***T ***T***AG GGC GAA GTA CGG G-BHQ-1(nucleotide Nos. 1760-1784) (bisulfite-converted nucleotides are highlighted by bold face and italics); P14 (CDKN2A/ARF)-F, 5′- ***T***TG GAG GCG GCG AGA A***T***A T-3′ (Genbank No. L41934, nucleotide Nos. 238-256); P14-R, 5′- CCC CGT ***A***A***A*** CCG CG***A*** ** ***A***AT ***A***-3′ (nucleotide Nos. 332-350); P14-probe, 6FAM-5′- CGG ***TT***C G***T***C GCG AGT GAG GGT T-3′ –BHQ-1 (nucleotide Nos. 299-320); WRN-F, 5′-G***T***A TCG ***TT***C GCG GCG ***TTT*** A***T***-3′ (Genbank No. AY442327, nucleotide Nos. 1827-1846); WRN-R, 5′-***A***CG ***AAA*** CCG ***A***T***A*** TCC GAA ***A***TC A -3′ (nucleotide Nos. 1887-1908) and WRN-probe, 6FAM-***TTT*** ** ***T***T***T*** ** ***T***TG CGG ***T***CG ***T***TG CGG G-BHQ-(nucleotide Nos. 1855-1876). The PCR condition was initial denaturation at 95°C for 10 min followed by 45 cycles of 95°C for 15 sec and 60°C for 1 min. A standard curve was made for each PCR plate by duplicated PCR amplifications for *COL2A1* on bisulfite-converted human genomic DNA at 4 different concentrations (in a 5-fold dilution series). The percentage of methylated reference (PMR, i.e., degree of methylation) at a specific locus was calculated by dividing the *GENE:COL2A1* ratio of template amounts in a sample by the *GENE:COL2A1* ratio of template amounts in *Sss*I-treated human genomic DNA (presumably fully methylated) and multiplying this value by 100. Methylation positivity was set as PMR≥4 as previously validated [31].

### Pyrosequencing to Measure LINE-1 Methylation

In order to accurately quantify relatively high LINE-1 methylation levels, we utilized Pyrosequencing as previously described [21]. LINE-1 methylation level measured by Pyrosequencing has been shown to correlate well with overall 5-methylcytosine level (i.e., genome-wide DNA methylation level) in tumor cells [32], [33].

### Immunohistochemistry for p53 and β-catenin

Tissue microarrays (TMAs) were constructed and immunohistochemistry for p53 and β-catenin was performed as previously described [19], [34]. Appropriate positive and negative controls were included in each run of immunohistochemistry. Cytoplasmic and nuclear β-catenin expression was recorded separately as either no expression (0), weak expression (1+), or moderate/strong expression (2+). The β-catenin activation score was calculated as the sum of nuclear score (0–2), cytoplasmic score (0–2) and membrane score (0 if membrane staining was positive, +1 if membrane expression was lost), as originally described by Jass et al. [35]. All immunohistochemically-stained slides were examined by one of the investigator (β-catenin by K.N.; p53 by S.O.) unaware of other data. Random samples of 402 and 118 tumors were re-examined for β-catenin and p53, respectively, by a second observer (β-catenin by S.O., p53 by K.N.) unaware of other data, and the concordances between the two observers were 0.83 for β-catenin (κ = 0.65, p<0.0001), and 0.87 for p53 (κ = 0.75, p<0.0001), indicating substantial agreement.

### Statistical Analysis

For cluster analysis of biomarkers including the 16 methylation markers, MSI, *KRAS* and *BRAF*, we utilized average linkage hierarchical clustering with a Euclidean distance metric as implemented in MeV (http://www.tm4.org) [36]. The chi square test was used to examine an association between CIMP and other categorical variables of interest. The t-test assuming unequal variances was performed to compare mean age and mean LINE-1 methylation level. The κ coefficient was calculated to assess agreement between each of the 16 markers (positive vs. negative) and the 16-marker CIMP panel (CIMP-high positive vs. negative).

To examine the relations of a given variable and CIMP-high, we utilized unconditional logistic regression models to calculate odds ratios (ORs) for CIMP-high, according to the status of the given variable, unadjusted and adjusted for age, sex, tumor location, stage, differentiation, LINE-1 methylation level, and status of MSI, *KRAS*, *BRAF*, p53 and β-catenin. To adjust for potential confounding, age and LINE-1 methylation level were used as continuous variables, and all of the other variables were used as categorical variables.

For age and LINE-1, we assessed non-linearity by the likelihood ratio test that compared a regression model including a quadratic (or cubic) term with a model excluding it. The likelihood ratio test showed that including the quadratic term did not significantly alter model fit (p = 0.86 for age, p = 0.078 for LINE-1), and that including the cubic term did not significantly alter model fit (p = 0.87 for age, p = 0.084 for LINE-1). We also examined the possibility of a non-linear relation between age (or LINE-1 methylation) and CIMP-high, non-parametrically with restricted cubic splines [37].

We dichotomized tumor location (proximal vs. distal), tumor differentiation (poor vs. well/moderate), signet ring cells (present vs. absent), MSI (high vs. non-MSI-high), p53 (positive vs. negative), *KRAS* (mutated vs. wild-type), *BRAF* (mutated vs. wild-type) and β-catenin (active vs. inactive). There were 3 categories for mucinous feature (0%, 1–49%, and ≥50%) in the initial main analysis (**Table 2**). We dichotomized mucinous feature (present vs. absent) in secondary stratified analyses and analyses of interactions, because multivariate ORs for CIMP-high were similar across the 1–49% mucinous and ≥50% mucinous categories (in reference to the non-mucinous category). There were 4 categories for stage (I, II, III and IV) in the initial main analysis (**Table 2**). We dichotomized tumor stage (I vs. II–IV) in secondary stratified analyses and analyses of interactions, because multivariate ORs for CIMP-high were similar across stage II–IV (in reference to stage I).

**Table 2:** Associations with CIMP-high in colorectal cancer by univariate and multivariate analyses.

Clinical, pathological, or molecular characteristics	Univariate OR (95% CI)	P value	OR adjusted for major confounder(s)	Major confounder(s) (in the order of strength of effect)	Multivariate OR (95% CI)	P value
Age (10-year increment)	1.78 (1.40–2.27)	<0.0001	2.53 (1.69–3.78)	*BRAF*, MSI	**3.16 (1.93–5.18)**	**<0.0001**
Female (vs. male)	1.79 (1.22–2.64)	0.003	-	-	1.96 (0.95–4.08)	0.071
Proximal (vs. distal)∧	13.3 (7.64–23.3)	<0.0001	5.58 (2.80–11.1)	MSI, *BRAF*	**3.39 (1.57–7.34)**	**0.002**
Tumor stage (vs. I)				tumor location, MSI, *BRAF*		
Stage II	4.42 (2.44–8.00)	<0.0001	2.53 (1.06–6.08)		1.83 (0.69–4.83)	0.23
Stage III	1.61 (0.83–3.12)	0.16	1.91 (0.72–5.12)		1.83 (0.63–5.28)	0.26
Stage IV	2.23 (1.06–4.71)	0.04	2.28 (0.78–6.65)		1.72 (0.53–5.62)	0.37
Poor differentiation	7.28 (4.50–11.8)	<0.0001	2.96 (1.42–6.19)	MSI, *BRAF*	**2.98 (1.15–7.69)**	**0.024**
Mucinous feature (vs. 0%)				MSI, *BRAF*		
1–49%	2.31 (1.46–3.64)	0.0003	1.72 (0.89–3.29)		1.53 (0.71–3.32)	0.28
≥50%	5.39 (3.37–8.63)	<0.0001	2.09 (1.03–4.24)		2.06 (0.81–5.22)	0.13
Signet ring cells, any	3.82 (2.18–6.71)	<0.0001	0.68 (0.32–1.41)	mucin, differentiation	0.98 (0.30–3.17)	0.97
MSI-high	41.9 (25.5–68.9)	<0.0001	27.6 (16.2–47.1)	tumor location	**21.2 (9.79–45.8)**	**<0.0001**
*BRAF* mutation	29.2 (18.0–47.6)	<0.0001	18.7 (11.0–31.7)	tumor location	**15.9 (6.60–38.4)**	**<0.0001**
*KRAS* mutation	0.25 (0.15–0.41)	<0.0001	0.71 (0.36–1.40)	*BRAF*, MSI	0.97 (0.44–2.10)	0.93
β-catenin activation*	0.14 (0.07–0.28)	<0.0001	0.21 (0.10–0.44)	tumor location	**0.21 (0.070–0.62)**	**0.005**
LINE-1 hypomethylation (30% decline)	0.20 (0.10–0.39)	<0.0001	0.30 (0.13–0.69)	MSI	**0.27 (0.094–0.75)**	**0.012**
p53 positivity	0.34 (0.22–0.52)	<0.0001	0.66 (0.39–1.13)	MSI	0.73 (0.35–1.51)	0.39

Multivariate logistic regression model includes all of the above variables. Note that female, tumor stage, signet ring cells, mucinous feature, *KRAS* and p53 are not significantly related with CIMP-high after adjusting for other variables.

* β-catenin activation is defined as β-catenin score ≥3. The β-catenin score is the sum of nuclear (0, 1+ or 2+), cytoplasmic (0, 1+ or 2+) and membrane (0 or 1+) scores as originally described by Jass et al.[35].

∧ Proximal colon includes cecum to transverse colon, and distal colon includes splenic flexure to rectum.

CI, confidence interval; CIMP, CpG island methylator phenotype; LINE-1, long interspersed nucleotide element-1; MSI, microsatellite instability; OR, odds ratio.

When there was missing information on tumor stage (12%), LINE-1 (3.9% missing), MSI (3.2% missing), p53 (1.3% missing), *KRAS* (2.3% missing) or *BRAF* (4.7% missing), we assigned a separate (“missing”) indicator variable and included those cases in the multivariate analysis models. We confirmed that excluding cases with a missing variable did not significantly alter results (data not shown). There was no missing information in other variables.

An association of each variable with CIMP-high was also assessed in strata of important clinical or molecular features, including age (<65 year old vs. ≥65 year old), sex, tumor location (proximal vs. distal), MSI status, and *BRAF* status. For stratified analysis, each multivariate logistic regression model included a variable of interest that was stratified by a given stratifying variable (e.g., age) and adjusted for all of the remaining variables (SAS codes available upon request).

An interaction was assessed by including the cross product term of a given variable (e.g., MSI) and another variable of interest in a regression model, and the likelihood ratio test compared a model including the cross product term with that excluding it. In addition to interactions of any given variable with MSI, location, age, sex and *BRAF*, we examined all possible remaining two-way interactions, and found no significant interactions (data not shown).

All statistical analyses except for clustering analysis used SAS version 9.1 (SAS Institute, Cary, NC). All p values were two-sided, and statistical significance was defined as p≤0.05. Nonetheless, multiple hypotheses testing was considered when interpreting the data, especially in examining multiple two-way interactions.

## Results

### Evaluation of 16 methylation markers

We obtained 904 colorectal cancer specimens and quantified DNA methylation in the 16 loci [*CACNA1G*, *IGF2*, *NEUROG1*, *RUNX3*, *SOCS1*, *CDKN2A* (p16), *CRABP1*, *MLH1*, *CHFR*, *HIC1*, *IGFBP3*, *MGMT*, MINT1, MINT31, p14 (*CDKN2A*/ARF), and *WRN*] by real-time PCR (MethyLight [30]) assays. The first 5 loci (up to *SOCS1*) were selected as good predictors of CIMP (CpG island methylator phenotype) by screening of 195 CpG islands [15]. The use of the first 8 loci (up to *MLH1*) as a CIMP-high diagnostic panel has been previously validated [24].

**Figure 1:**
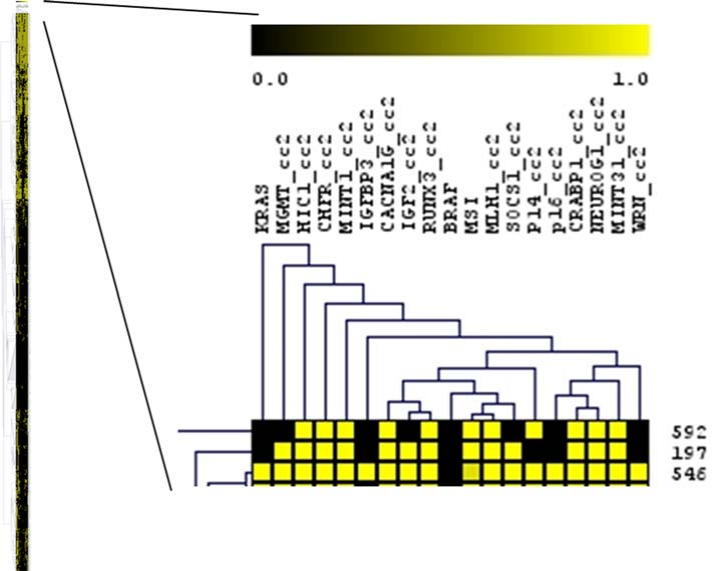
Hierarchical clustering analysis of 16 methylation makers, MSI, *KRAS* and *BRAF* in colorectal cancers. Horizontal and vertical axes represent markers and cases, respectively. The expanded view of clustering tree for the markers is shown on the right. The 8 markers in our CIMP-high diagnostic panel (*CACNA1G*, *IGF2*, *RUNX3*, *MLH1*, *SOCS1*, *CDKN2A* (p16), *CRABP1* and *NEUROG1*) are clustered closely, supporting that these markers are good CIMP-high markers. Also note the close relationship between MSI, *BRAF* and the 8 CIMP-high markers. *KRAS* mutation is not clustered with any of the methylation markers analyzed, suggesting that *KRAS* mutation (which is associated with CIMP-low [24], [29]; see Supplemental Figure) is probably associated with a random methylation pattern.

To evaluate 16 methylation markers in an unbiased fashion, we conducted an unsupervised hierarchical clustering analysis of the 16 methylation markers and status of MSI (microsatellite instability), and *KRAS* and *BRAF* oncogenes, using 860 tumors with all of these results available (**Figure 1**). The 8 CIMP-high markers (*CACNA1G*, *CDKN2A* (p16), *CRABP1*, *IGF2*, *MLH1*, *NEUROG1*, *RUNX3* and *SOCS1*) were generally clustered together, indicating good concordance of methylation patterns in these markers and supporting these 8 markers as good CIMP-high markers. In addition, p14, MINT31 and *WRN* were also clustered with the 8 markers. The other 5 methylation markers (*MGMT*, *HIC1*, *CHFR*, MINT1 and *IGFBP3*) were not clustered closely with each other or the 8 markers. The *BRAF* and MSI variables, which have been known to be associated with CIMP-high [15], [18], [24], were also clustered together with these 8 markers, indicating tight associations with CIMP-high. Notably, *KRAS* mutation was not clustered with any of the methylation markers, suggesting its association with a random methylation pattern (particularly in CIMP-low tumors which have been associated with *KRAS* mutation [29]; see also **Supplemental** **Figure S1**). We used clustering analysis only for the examination of marker clustering, but not for tumor classification. That was because clustering of markers was very stable with the large number of tumors (i.e., excluding a few tumors did not substantially influence results) while tumor classification by clustering analysis based on the 16 markers was not stable.

To describe performance of each of the 16 markers in an unbiased way, we calculated κ coefficient (for agreement statistics), sensitivity and specificity of each maker for CIMP-high diagnosis determined by the 16 markers (**Supplemental** **Table S1**). The cutoff for CIMP-high was set as ≥11/16 or ≥10/16 methylated markers based on the distribution of *KRAS* and *BRAF* mutations (**Supplemental Figure**), and on the previous findings that CIMP-high is associated with *BRAF* mutation and CIMP-low is associated with *KRAS* mutation [24], [29]. Sensitivity and specificity of each marker reflected overall concordance of a methylation pattern with the remaining 15 markers. It was evident that performance of the 8 CIMP-panel markers (*CACNA1G*, *CDKN2A*, *CRABP1*, *IGF2*, *MLH1*, *NEUROG1*, *RUNX3* and *SOCS1*) was generally good. The κ coefficient was greater than 0.5 for all of these 8 markers. *RUNX3* was the single best marker for CIMP-high diagnosis. Among the other 8 markers (*CHFR*, *HIC1*, *IGFBP3*, *MGMT*, MINT1, MINT31, p14 and *WRN*), only MINT31 and p14 consistently showed the κ coefficient greater than 0.5, and good sensitivity/specificity. This was in agreement with clustering analysis, which showed that MINT31 and p14 clustered with the 8 CIMP-panel markers.

We also compared the all-16-marker panel with the 8-marker CIMP panel. Using the 8-maker panel, or the 16-maker panel, CIMP-high was defined as ≥6/8 or ≥11/16 methylated markers, respectively. Among the 904 cases, 879 cases (97.2%) showed concordant diagnosis of CIMP status between the 16-marker panel and the 8-marker panel (κ = 0.89, p<0.0001). When the 16-marker CIMP panel was used, the associations of CIMP-high with clinical and molecular features were very similar to the CIMP-high associations by the 8-marker CIMP panel (data not shown). We also confirmed a high degree of agreement (98.6% concordant; κ = 0.94) between the 8-marker panel and the 5-marker panel described by Weisenberger et al. [15]. Thus, in subsequent analyses, we used the 8-marker CIMP panel which we had extensively validated [24].

### CIMP-high in colorectal cancer

We assessed clinical, pathologic and molecular features of CIMP-high colorectal cancer (**Table 1**). By univariate analysis, CIMP-high was associated with female sex, older age, proximal location, poor differentiation, mucin, signet ring cells, MSI-high and *BRAF* mutation, and inversely with stage I, *KRAS* mutation, LINE-1 hypomethylation, positive p53, and active β-catenin (all p<0.004).

**Figure 2:**
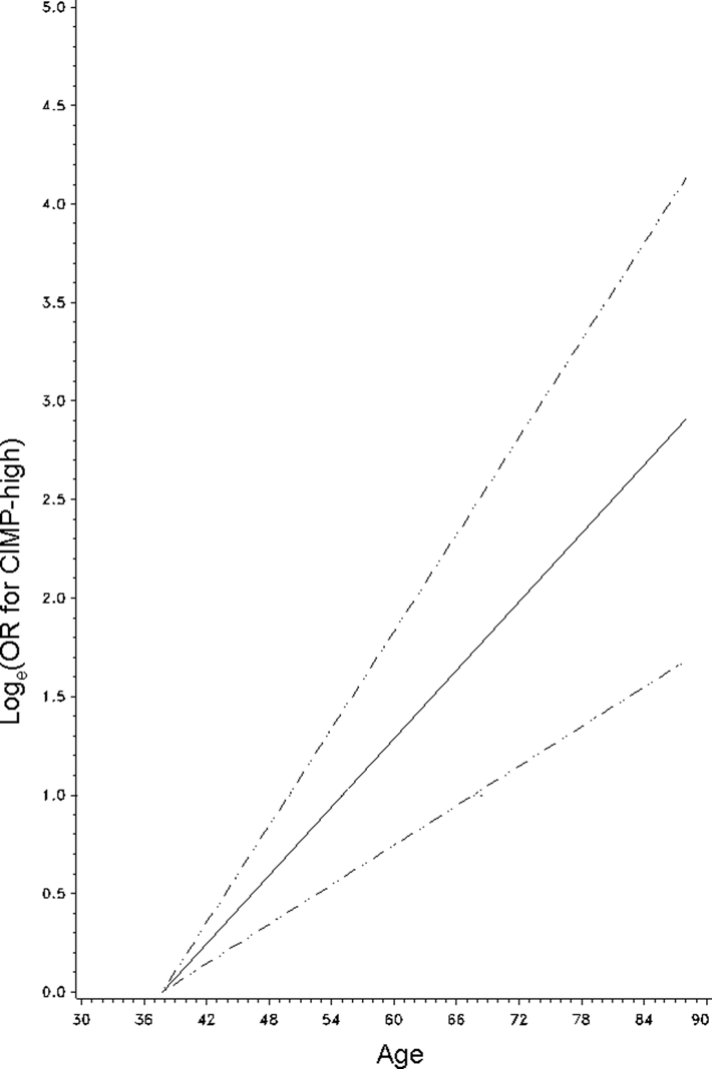
Smoothing spline for the age/CIMP-high association. Log_e_(OR for CIMP-high) (y axis) according to age (x axis) is shown (with young age as a referent). Broken lines indicate 95% confidence interval. Note the linear relation between age and CIMP-high. CIMP, CpG island methylator phenotype; OR, odds ratio.

**Figure 3:**
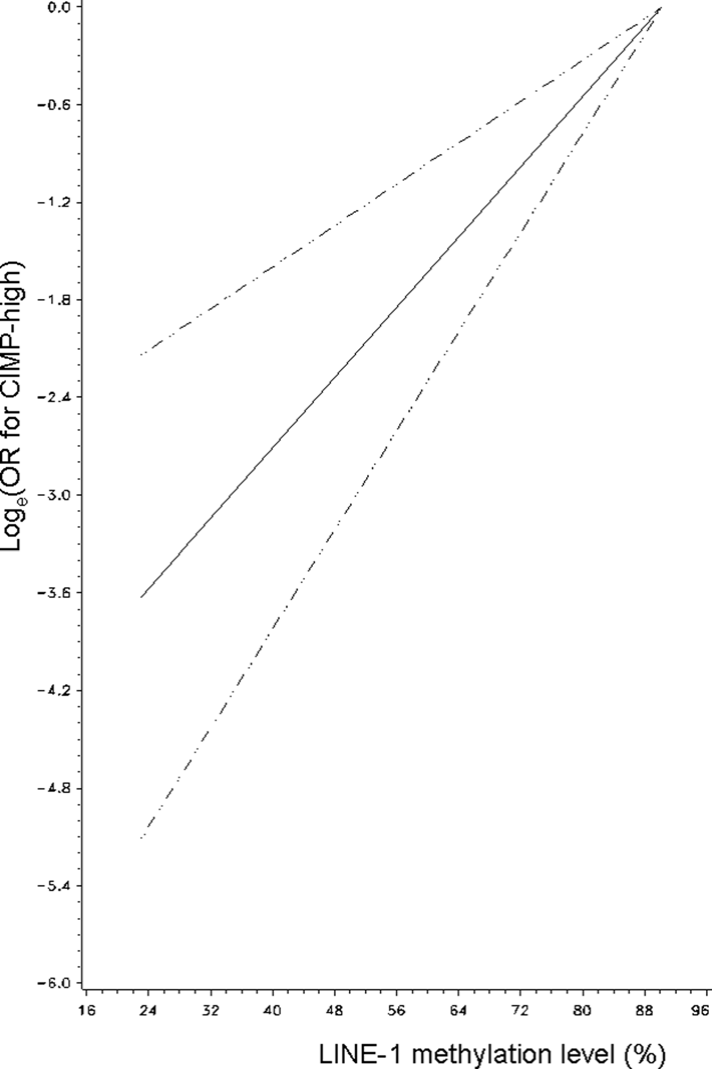
Smoothing spline for the LINE-1/CIMP-high association. Log_e_(OR for CIMP-high) (y axis) according to LINE-1 methylation (x axis) is shown (with high-level LINE-1 methylation as a referent). Broken lines indicate 95% confidence interval. Note the inverse linear relation between LINE-1 hypomethylation and CIMP-high. CIMP, CpG island methylator phenotype; LINE-1, long interspersed nucleotide element-1; OR, odds ratio.

Age was linearly associated with CIMP-high in logistic regression analysis (p for trend <0.0001). We did not show significant non-linearity by the likelihood ratio test that compared a model including a quadratic (or cubic) term with a model excluding it (p>0.85). Likewise, LINE-1 hypomethylation was inversely linearly associated with CIMP-high (p for trend <0.0001), and there was no significant non-linearity by the likelihood ratio test, using a quadratic (or cubic) term (p≥0.078). Non-parametric restricted cubic splines also supported a linear relation between age and CIMP-high (**Figure 2**) and an inverse linear relation between LINE-1 hypomethylation and CIMP-high (**Figure 3**).

**Figure 4:**
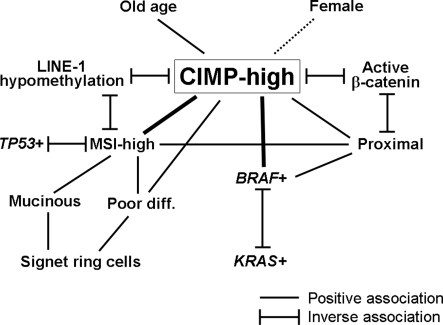
Summary of associations of CIMP-high with clinical and molecular features. The broken line indicates the relatively weak association.

In multivariate logistic regression analysis, CIMP-high was significantly associated with older age, proximal location, poor differentiation, MSI-high and *BRAF* mutation, and inversely with active β-catenin and LINE-1 hypomethylation (**Table 2**). However, all of the other features (female, stage, mucin, signet ring cells, *KRAS* and p53) were no longer significantly associated with CIMP-high in multivariate analysis. We further examined for potential confounders in the association of each variable with CIMP-high. Except for sex, all of the other variables showed substantial changes in odds ratio (OR) for the association with CIMP-high after adjusting for MSI, *BRAF* and/or tumor location (or other variables) (**Table 2**). These results indicated the existence of complex relations between clinical and molecular features (including CIMP) in colorectal cancer, which are summarized in **Figure 4**.

### Associations with CIMP-high in strata of MSI

Molecular classification by MSI status is increasingly important in colorectal cancer [38]–[40]. Thus, we examined the relations of clinical and tumoral variables with CIMP-high in MSI-high tumors and non-MSI-high tumors (**Table 3**). Older age, proximal location and *BRAF* mutation were significantly associated with CIMP-high in both MSI-high and non-MSI-high tumors. In contrast, the relations of CIMP-high with poor differentiation, *KRAS* mutation and LINE-1 hypomethylation appeared to be different according to MSI status (p for interaction <0.005). CIMP-high was associated with poor differentiation and inversely with *KRAS* mutation in MSI-high tumors, but not in non-MSI-high tumors. LINE-1 hypomethylation was inversely associated with CIMP-high in non-MSI-high tumors, but not in MSI-high tumors.

**Table 3:** Associations with CIMP-high in MSI-high and non-MSI-high colorectal cancers.

Clinical, pathological, or molecular characteristics	P for interaction with MSI	Among non-MSI-high tumors		Among MSI-high tumors	
		Multivariate OR (95% CI)	P value	Multivariate OR (95% CI)	P value
Age (10-year increment)	0.033	2.12 (1.23–3.65)	0.007	6.14 (2.49–15.1)	<0.0001
Female (vs. male)	0.46	1.41 (0.57–3.47)	0.45	2.03 (0.69–6.06)	0.20
Proximal (vs. distal)∧	0.33	3.01 (1.22–7.40)	0.016	7.17 (1.74–29.5)	0.006
Tumor stage II–IV (vs. I)	0.064	0.97 (0.35–2.68)	0.95	4.92 (1.18–20.5)	0.029
Poor differentiation	**0.002**	0.84 (0.21–3.30)	0.80	**25.6 (3.43–191)**	**0.002**
Mucinous feature, any	0.29	2.02 (0.86–4.74)	0.11	0.95 (0.30–2.99)	0.93
Signet ring cells, any	0.22	1.65 (0.44–6.17)	0.46	0.42 (0.065–2.71)	0.36
*BRAF* mutation	0.21	11.7 (4.34–31.6)	<0.0001	49.8 (4.71–527)	0.001
*KRAS* mutation	**0.004**	2.24 (0.81–6.17)	0.12	**0.17 (0.039–0.78)**	**0.022**
p53 positivity	0.65	0.72 (0.31–1.70)	0.46	0.50 (0.12–2.00)	0.33
LINE-1 hypomethylation (30% decline)	**0.002**	**0.095 (0.026–0.34)**	**0.0003**	3.20 (0.47–22.0)	0.24
β-catenin activation*	0.18	0.32 (0.097–1.04)	0.058	0.063 (0.008–0.51)	0.009

Each multivariate logistic regression model assesses a variable of interest (stratified by MSI status in a given model), adjusting for all of the above remaining variables. An interaction was assessed by the likelihood ratio test that compares a model including a cross product term (of the MSI variable and another variable of interest) with a model excluding it.

* β-catenin activation is defined as β-catenin score ≥3, where the β-catenin score is the sum of nuclear (0, 1+ or 2+), cytoplasmic (0, 1+ or 2+) and membrane (0 or 1+) scores as originally described by Jass et al.[35].

∧ Proximal colon includes cecum to transverse colon, and distal colon includes splenic flexure to rectum.

CI, confidence interval; CIMP, CpG island methylator phenotype; LINE-1, long interspersed nucleotide element-1; MSI, microsatellite instability; OR, odds ratio.

### Associations with CIMP-high in strata of tumor location

There is accumulating evidence for a molecular difference between proximal and distal colorectal cancers [38], [41]. Therefore, we examined the relations of clinical and tumoral variables with CIMP-high in proximal tumors and distal tumors (**Table 4**). The relations of CIMP-high with the variables did not appear to differ according to tumor location (all p for interaction >0.23).

**Table 4:** Associations with CIMP-high in proximal and distal colorectal cancers.

Clinical, pathological, or molecular characteristics	P for interaction with location	Among proximal tumors∧		Among distal tumors∧	
		Multivariate OR (95% CI)	P value	Multivariate OR (95% CI)	P value
Age (10-year increment)	0.32	2.62 (1.58–4.34)	0.0002	4.62 (1.61–13.2)	0.004
Female (vs. male)	0.71	1.93 (0.87–4.30)	0.11	1.21 (0.32–4.58)	0.78
Tumor stage II–IV (vs. I)	0.31	2.48 (0.79–7.79)	0.12	1.00 (0.24–4.15)	0.99
Poor differentiation	0.46	2.54 (0.91–7.10)	0.075	5.81 (0.89–37.8)	0.066
Mucinous feature, any	0.24	1.25 (0.58–2.72)	0.57	3.14 (0.79–12.5)	0.10
Signet ring cells, any	0.56	0.86 (0.25–2.94)	0.81	2.01 (0.18–22.6)	0.57
MSI-high	0.33	31.0 (13.2–73.2)	<0.0001	14.3 (3.28–62.5)	0.0004
*BRAF* mutation	0.56	13.7 (5.21–36.1)	<0.0001	22.8 (4.78–109)	<0.0001
*KRAS* mutation	0.39	1.20 (0.48–2.99)	0.70	0.57 (0.13–2.44)	0.44
p53 positivity	0.37	0.89 (0.39–2.06)	0.79	0.37 (0.083–1.63)	0.19
LINE-1 hypomethylation (30% decline)	0.26	0.19 (0.055–0.63)	0.007	0.65 (0.098–4.24)	0.65
β-catenin activation*	-#	0.35 (0.11–1.13)	0.078	0#	-#

Each multivariate logistic regression model assesses a variable of interest (stratified by tumor location in a given model), adjusting for all of the above remaining variables. An interaction was assessed by the likelihood ratio test that compares a model including a cross product term (of the location variable and another variable of interest) with a model excluding it.

* β-catenin activation is defined as β-catenin score ≥3, where the β-catenin score is the sum of nuclear (0, 1+ or 2+), cytoplasmic (0, 1+ or 2+) and membrane (0 or 1+) scores as originally described by Jass et al.[35].

∧ Proximal colon includes cecum to transverse colon, and distal colon includes splenic flexure to rectum.

# Because there was no CIMP-high β-catenin-active tumors in distal colon, stable p for interaction and 95% CI for OR could not be estimated.

CI, confidence interval; CIMP, CpG island methylator phenotype; LINE-1, long interspersed nucleotide element-1; MSI, microsatellite instability; OR, odds ratio.

### Associations with CIMP-high in other stratified analyses

We examined the relations of clinical and tumoral variables with CIMP-high in strata of sex, age (<65 year old vs. ≥65 year old) and *BRAF* status. Considering multiple hypotheses testing (12-hypotheses testing each), the effect of the variables did not appear to significantly differ according to age (all p for interaction >0.03) and sex (all p for interaction >0.02). Notably, the effect of LINE-1 hypomethylation did appear to differ according to *BRAF* status (p for interaction = 0.001) (**Table 5**). A significant inverse association of LINE-1 hypomethylation with CIMP-high was present in *BRAF*-mutated tumors [adjusted OR = 0.022; 95% confidence interval (CI), 0.003–0.17], but not in *BRAF*-wild-type tumors (adjusted OR = 0.87; 95% CI, 0.25–3.06).

**Table 5:** Associations with CIMP-high in *BRAF*-wild-type and *BRAF*-mutated colorectal cancers.

Clinical, pathological, or molecular characteristics	P for interaction with *BRAF*	Among *BRAF*-wild-type tumors		Among *BRAF*-mutated tumors	
		Multivariate OR (95% CI)	P value	Multivariate OR (95% CI)	P value
Age (10-year increment)	0.13	3.60 (2.02–6.43)	<0.0001	1.80 (0.81–4.02)	0.15
Female (vs. male)	0.23	2.27 (1.01–5.09)	0.047	0.70 (0.14–3.51)	0.67
Proximal (vs. distal)∧	0.56	3.87 (1.57–9.50)	0.003	2.33 (0.52–10.4)	0.27
Tumor stage II–IV (vs. I)	0.043	2.79 (0.92–8.45)	0.69	0.37 (0.070–1.94)	0.24
Poor differentiation	0.27	3.84 (1.27–11.6)	0.017	1.43 (0.32–6.49)	0.64
Mucinous feature, any	0.98	1.56 (0.69–3.56)	0.29	1.54 (0.45–5.30)	0.50
Signet ring cells, any	0.25	0.67 (0.15–3.09)	0.61	2.55 (0.40–16.3)	0.32
MSI-high	0.21	21.6 (9.56–48.6)	<0.0001	82.6 (8.48–804)	0.0001
*KRAS* mutation	0.37	1.02 (0.44–2.35)	0.96	0.18 (0.005–6.69)	0.35
p53 positivity	0.52	0.55 (0.21–1.40)	0.21	0.90 (0.26–3.06)	0.86
LINE-1 hypomethylation (30% decline)	**0.001**	0.87 (0.25–3.06)	0.83	0.022 (0.003–0.17)	0.0003
β-catenin activation*	0.44	0.20 (0.052–0.76)	0.018	0.49 (0.076–3.21)	0.46

Each multivariate logistic regression model assesses a variable of interest (stratified by *BRAF* status in a given model), adjusting for all of the above remaining variables. An interaction was assessed by the likelihood ratio test that compares a model including a cross product term (of the *BRAF* variable and another variable of interest) with a model excluding it.

* β-catenin activation is defined as β-catenin score ≥3, where the β-catenin score is the sum of nuclear (0, 1+ or 2+), cytoplasmic (0, 1+ or 2+) and membrane (0 or 1+) scores as originally described by Jass et al.[35].

∧ Proximal colon includes cecum to transverse colon, and distal colon includes splenic flexure to rectum.

CI, confidence interval; CIMP, CpG island methylator phenotype; LINE-1, long interspersed nucleotide element-1; MSI, microsatellite instability; OR, odds ratio.

We also examined all of the remaining potential two-way interactions by the available clinical and tumoral variables, and found no significant interactions with regard to the associations with CIMP-high (data not shown).

## Discussion

In this study utilizing a large sample size, we evaluated 16 methylation makers in an unbiased fashion. The 16 methylation markers included the 5 markers (*CACNA1G*, *IGF2*, *NEUROG1*, *RUNX3* and *SOCS1*) that were selected by screening of 195 CpG islands [15] and further validated to be included in the CIMP-high diagnostic panel (the above 5 plus *CDKN2A*, *CRABP1* and *MLH1*) [24]. By unsupervised hierarchical clustering analysis, the 5 methylation markers were clustered with each other as well as with MSI (microsatellite instability) and *BRAF* mutation. Analysis of κ coefficient, sensitivity and specificity also demonstrated good performance of the 5 methylation markers with generally concordant methylation pattern. Utilizing the validated CIMP panel, we have deciphered the complex relations of CIMP-high with various clinical, pathologic and molecular features in colorectal cancer. Our data provide a rationale for the of the validated CIMP-specific methylation marker panel.

This study is the first extensive investigation to compare the 5 new CIMP-high markers [15] with MINT1, MINT31 and other CpG islands, using a large sample size. Performance of the 5 new markers (*CACNA1G*, *IGF2*, *NEUROG1*, *RUNX3*, and *SOCS1*), *CRABP1* and *MLH1* was consistently superior to that of *WRN*, MINT1, *CHFR*, *IGFBP3*, *HIC1* and *MGMT*. MINT31, *CDKN2A*, and p14 showed intermediate performance characteristics, and in hierarchical clustering analysis, were generally clustered with the new 5 CIMP markers, MSI and *BRAF* mutation. We have provided valuable data for standardization of methylation markers for the detection of CIMP-high in colorectal cancer.

Studying epigenetic and genetic aberrations is important in cancer research [42]–[46]. We used quantitative PCR assays (MethyLight [30]) to determine the degree of DNA methylation, which is robust enough to reproducibly differentiate low-level methylation from high-level methylation [31]. Our resource of a large colorectal cancer sample obtained from two large prospective cohorts (representing a relatively unbiased sample compared to a single-hospital-based sample) has provided a sufficient power to evaluate the 16 methylation markers, and to simultaneously assess independent relations of CIMP-high with multiple clinical and tumoral molecular variables.

Interestingly, unsupervised clustering analysis using a large number of tumors revealed that *KRAS* mutation was not clustered with any of the 16 methylation markers. However, as shown in our previous studies [24], [29], *KRAS* mutation was more common in CIMP-low tumors compared to CIMP-high and CIMP-0 tumors. Although these findings appeared to be discrepant, we believe that *KRAS* mutation is perhaps associated with a random pattern of CpG island methylation, indicated by the non-clustering phenomenon in clustering analysis. In contrast, our clustering analysis has clearly shown that *BRAF* mutation is clustered with CIMP-high specific markers, indicating that *BRAF* mutation is perhaps associated with a non-random pattern of CpG island methylation.

Previous studies identified various factors associated with CIMP-high, including old age, female, proximal location, poor differentiation, mucin, signet ring cells, MSI-high, *BRAF* mutation, wild-type *KRAS*, inactive β-catenin, wild-type *APC*, high LINE-1 methylation level, and wild-type *TP53* [9]–[19], [21], [47], [48]. However, many of these factors are interrelated. Thus, in order to properly decipher the relations with CIMP-high, it is necessary to use a large number of tumors, determine a number of molecular features, and perform comprehensive biostatistical analysis. We were able to utilize a large colorectal cancer sample that has been examined for multiple molecular events, and appropriate biostatistical methods. **Figure 3** summarizes our current knowledge on the associations of clinical, pathologic and molecular features including CIMP in colorectal cancer. It is very important to keep in mind these relations, when analyzing the association between any of these factors and an outcome (e.g., molecular changes in colorectal cancer, patient mortality, etc.). These factors may confound the relationship of interest. Indeed, we have demonstrated confounding effects of MSI, *BRAF* and tumor location in a number of the associations in **Table 2**. In particular, signet ring cells, *KRAS* and p53 were no longer associated with CIMP-high after adjusting for the confounders.

We have shown that the relations of CIMP-high with tumor differentiation, *KRAS* mutation and LINE-1 hypomethylation appear to differ according to MSI status. MSI is a major molecular classifier in colorectal cancer [38]–[40]. MSI-high tumors have been shown to exhibit widespread mutations in nucleotide repeat sequences such as those in *TGFBR2* and *BAX* [49], [50]. Thus, it is likely that overall genomic changes in MSI-high tumors are dissimilar to those of non-MSI-high tumors. That may explain why there are some pathologic and molecular features that are differentially associated with CIMP-high according to MSI status.

In summary, using the 16 methylation markers and a large population-based sample, we have evaluated performance of each of the 16 methylation markers in an unbiased fashion. Our current study provides valuable data for standardization of the use of CIMP-high-specific markers. Using the validated CIMP-specific methylation marker panel, we have comprehensively analyzed the clinical, pathologic and molecular features of CIMP-high colorectal cancer by comprehensive biostatistical methods. We have provided the rationale to use the validated CIMP-high-specific methylation marker panel in clinical and research settings. Further studies are necessary to elucidate fundamental molecular defects that lead to CIMP-high colorectal cancer.

## Supporting Information

Figure S1 Distribution of colorectal cancers according to the number of methylated markers and KRAS/BRAF mutational status. Note that KRAS mutation is associated with CIMP-low (rather than CIMP-high and CIMP-negative), in agreement with studies using more limited CIMP-specific methylation markers [24], [29].
(0.12 MB TIF)

Table S1 Markers are listed in the order of the kappa coefficient. * Sensitivity of each marker is defined as “[the number of CIMP-high cases positive for a given marker] / [the number of all CIMP-high cases]”. ∧ Specificity of each marker is defined as “[the number of non-CIMP-high cases negative for a given marker] / [the number of all non-CIMP-high cases]”.
(0.15 MB DOC)
