# Supplementary figures and images for: Comprehensive Biostatistical Analysis of CpG Island Methylator Phenotype in Colorectal Cancer Using a Large Population-Based Sample
Source: PLoS One. 2008 Nov 12;3(11):e3698. doi: 10.1371/journal.pone.0003698 (PMC2579485; doi:10.1371/journal.pone.0003698)

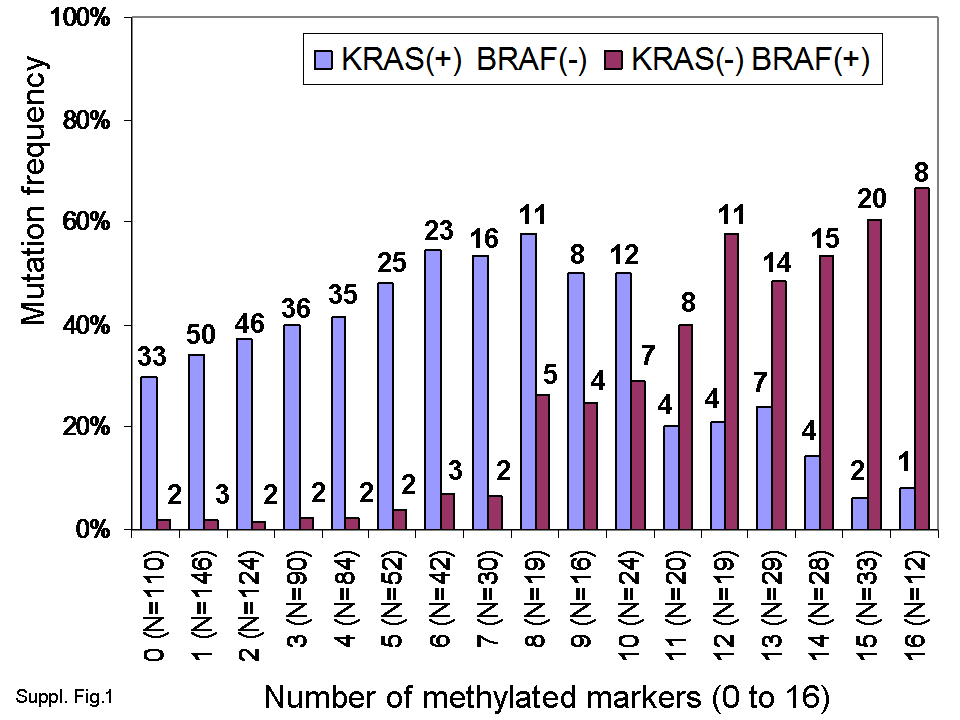

Supplement: Figure S1 — Distribution of colorectal cancers according to the number of methylated markers and KRAS/BRAF mutational status. Note that KRAS mutation is associated with CIMP-low (rather than CIMP-high and CIMP-negative), in agreement with studies using more limited CIMP-specific methylation markers [24], [29]. (0.12 MB TIF) [file pone.0003698.s001.tif]
