# Supplementary material for: Comprehensive Biostatistical Analysis of CpG Island Methylator Phenotype in Colorectal Cancer Using a Large Population-Based Sample
Source: PLoS One. 2008 Nov 12;3(11):e3698. doi: 10.1371/journal.pone.0003698 (PMC2579485; doi:10.1371/journal.pone.0003698)
Supplement: Table S1 — Markers are listed in the order of the kappa coefficient. * Sensitivity of each marker is defined as “[the number of CIMP-high cases positive for a given marker] / [the number of all CIMP-high cases]”. ∧ Specificity of each marker is defined as “[the number of non-CIMP-high cases negative for a given marker] / [the number of all non-CIMP-high cases]”. (0.15 MB DOC) [file pone.0003698.s002.doc]

Supplemental Table. Unbiased evaluation of each methylation marker for CIMP-high diagnosis

|  |  |  | CIMP-high (≥11/16 methylated markers) | | |  | CIMP-high (≥10/16 methylated markers) | | |
| --- | --- | --- | --- | --- | --- | --- | --- | --- | --- |
| Marker |  | Total No. | κ coefficient |  |  |  | κ coefficient |  |  |
|  |  |  |  | Positive (sensitivity)* | Negative (specificity)^ |  |  | Positive (sensitivity)* | Negative (specificity)^ |
|  |  |  |  |  |  |  |  |  |  |
|  |  | 904 |  | 142 | 762 |  |  | 166 | 738 |
|  |  |  |  |  |  |  |  |  |  |
|  |  |  |  |  |  |  |  |  |  |
| *RUNX3* | (+) | 179 | 0.79 | 133 (93.7%) | 46 |  | 0.84 | 150 (90.4%) | 29 |
|  | (-) | 725 |  | 9 | 716 (94.0%) |  |  | 16 | 709 (96.1%) |
|  |  |  |  |  |  |  |  |  |  |
| *IGF2* | (+) | 211 | 0.72 | 137 (96.5%) | 74 |  | 0.78 | 156 (94.0%) | 55 |
|  | (-) | 693 |  | 5 | 688 (90.3%) |  |  | 10 | 683 (92.5%) |
|  |  |  |  |  |  |  |  |  |  |
| *CACNA1G* | (+) | 195 | 0.72 | 130 (91.5%) | 65 |  | 0.75 | 145 (87.3%) | 50 |
|  | (-) | 709 |  | 12 | 697 (91.5%) |  |  | 21 | 688 (93.2%) |
|  |  |  |  |  |  |  |  |  |  |
| *MLH1* | (+) | 112 | 0.68 | 92 (64.8%) | 20 |  | 0.63 | 95 (57.2%) | 17 |
|  | (-) | 792 |  | 50 | 742 (97.4%) |  |  | 71 | 721 (97.7%) |
|  |  |  |  |  |  |  |  |  |  |
| *NEUROG1* | (+) | 268 | 0.57 | 135 (95.1%) | 133 |  | 0.62 | 153 (92.2%) | 115 |
|  | (-) | 636 |  | 7 | 629 (82.5%) |  |  | 13 | 623 (84.4%) |
|  |  |  |  |  |  |  |  |  |  |
| *SOCS1* | (+) | 151 | 0.57 | 94 (66.2%) | 57 |  | 0.56 | 101 (60.8%) | 50 |
|  | (-) | 753 |  | 48 | 705 (92.5%) |  |  | 65 | 688 (93.2%) |
|  |  |  |  |  |  |  |  |  |  |
| *CRABP1* | (+) | 285 | 0.56 | 139 (97.9%) | 146 |  | 0.62 | 159 (95.8%) | 126 |
|  | (-) | 619 |  | 3 | 616 (80.8%) |  |  | 7 | 612 (82.9%) |
|  |  |  |  |  |  |  |  |  |  |
| MINT31 | (+) | 299 | 0.54 | 140 (98.6%) | 159 |  | 0.60 | 161 (97.0%) | 138 |
|  | (-) | 605 |  | 2 | 603 (79.1%) |  |  | 5 | 600 (81.3%) |
|  |  |  |  |  |  |  |  |  |  |
| *CDKN2A* (p16) | (+) | 269 | 0.51 | 126 (88.7%) | 143 |  | 0.56 | 143 (86.1%) | 126 |
|  | (-) | 635 |  | 16 | 619 (81.2%) |  |  | 23 | 612 (82.9%) |
|  |  |  |  |  |  |  |  |  |  |
| P14 (*CDKN2A* / ARF) | (+) | 180 | 0.51 | 96 (67.6%) | 84 |  | 0.51 | 105 (63.3%) | 75 |
|  | (-) | 724 |  | 46 | 678 (89.0%) |  |  | 61 | 663 (89.8%) |
|  |  |  |  |  |  |  |  |  |  |
| *WRN* | (+) | 334 | 0.46 | 137 (96.5%) | 197 |  | 0.52 | 159 (95.8%) | 175 |
|  | (-) | 570 |  | 5 | 565 (74.1%) |  |  | 7 | 563 (76.3%) |
|  |  |  |  |  |  |  |  |  |  |
| MINT1 | (+) | 316 | 0.40 | 121 (85.2%) | 195 |  | 0.40 | 131 (78.9%) | 185 |
|  | (-) | 588 |  | 21 | 567 (74.4%) |  |  | 35 | 553 (74.9%) |
|  |  |  |  |  |  |  |  |  |  |
| *CHFR* | (+) | 387 | 0.36 | 134 (94.4%) | 253 |  | 0.38 | 150 (90.4%) | 237 |
|  | (-) | 517 |  | 8 | 509 (66.8%) |  |  | 16 | 501 (67.9%) |
|  |  |  |  |  |  |  |  |  |  |
| *IGFBP3* | (+) | 238 | 0.35 | 91 (64.1%) | 147 |  | 0.39 | 106 (63.9%) | 132 |
|  | (-) | 666 |  | 51 | 615 (80.7%) |  |  | 60 | 606 (82.1%) |
|  |  |  |  |  |  |  |  |  |  |
| *HIC1* | (+) | 467 | 0.24 | 128 (90.1%) | 339 |  | 0.27 | 148 (89.2%) | 319 |
|  | (-) | 437 |  | 14 | 423 (55.5%) |  |  | 18 | 419 (56.7%) |
|  |  |  |  |  |  |  |  |  |  |
| *MGMT* | (+) | 346 | 0.12 | 78 (54.9%) | 268 |  | 0.13 | 89 (53.6%) | 257 |
|  | (-) | 558 |  | 64 | 494 (64.8%) |  |  | 77 | 481 (65.2%) |
|  |  |  |  |  |  |  |  |  |  |

Markers are listed in the order of the κ coefficient.

* Sensitivity of each marker is defined as “[the number of CIMP-high cases positive for a given marker] / [the number of all CIMP-high cases]”. ^ Specificity of each marker is defined as “[the number of non-CIMP-high cases negative for a given marker] / [the number of all non-CIMP-high cases]”.
